# Safety of SARS-CoV-2 vaccine in patients with autoimmune neurological conditions: A systematic review and meta-analysis

**DOI:** 10.1016/j.heliyon.2023.e23944

**Published:** 2023-12-23

**Authors:** Fan Ning, Xiang-qi Cao, Qing-qing Wang, Zhu-yi Li, Zhe Ruan, Ting Chang

**Affiliations:** Department of Neurology, Tangdu Hospital, The Fourth Military Medical University, Xi'an, China

**Keywords:** SARS-CoV-2 vaccine, Autoimmune neurological conditions, Safety, Vaccination hesitancy

## Abstract

**Introduction:**

Risk of adverse effects and exacerbation in autoimmune neurological conditions (ANC)are frequently cited reasons for COVID-19 vaccine hesitancy. This study evaluates the ANC safety of COVID-19 vaccines in the real world.

**Methods:**

Electronic databases were searched to identify studies reporting the use of the COVID-19 vaccine in ANC. We selected studies that provided data on adverse effects and worsening conditions related to ANC after vaccination. The pooled incidence rates for various adverse effects, stratified for the disease category, dosage, and type of vaccine, were estimated.

**Results:**

Twenty-eight studies (31 vaccination cohorts) were included. The pooled incidence rate of general adverse events was 0.35 (95%CI, 0.27–0.43, I^2^ = 100 %). The pooled incidence rates of local injection reaction, fatigue, weakness, myalgia, fever, headache, and chills were 0.27 (0.18–0.36, I^2^ = 98 %), 0.16(0.11–0.21, I^2^ = 93 %), 0.15(0.00–0.31, I^2^ = 97 %), 0.13(0.08–0.19, I^2^ = 97 %), 0.11(0.07–0.15, I^2^ = 95 %), 0.11(0.07–0.16, I^2^ = 97 %), and 0.09 (0.03–0.16, I^2^ = 96 %), respectively. The pooled incidence rate of exacerbation adverse events was 0.05 (95%CI, 0.04–0.07, I^2^ = 84 %).

**Conclusion:**

According to available evidence, the administration of COVID-19 vaccines in individuals with autoimmune neurological disorders seems well-tolerated, with few reports of adverse events. Furthermore, exacerbation of autoimmune neurological conditions following vaccination appears to be infrequent.

## 1 Introduction

Since December 2019, COVID-19 infection caused by severe acute respiratory syndrome type 2 coronavirus (SARS-CoV-2) has rapidly spread worldwide, posing significant challenges to public health systems in various countries [1]. SARS-CoV-2 has been undergoing multiple rounds of variation since the outbreak [2]. As of March 31, 2023, it has caused more than 6.8 million deaths worldwide, and the total medical costs and other economic losses caused by the prevention and treatment of COVID-19 are unprecedented [3]. In addition, long-term chronic epidemics of the disease and various anti-epidemic measures have caused many psychological and social problems among the population, exacerbating social instability, particularly in less developed countries [4]. Presently, there are few specific drugs against this highly contagious ribonucleic acid (RNA) virus, and widespread vaccination of the population is considered one of the effective interventions to reduce morbidity and mortality substantially and end the virus epidemic [5]. As of March 31, 2022, more than 13 billion vaccine doses have been administered worldwide [3]. Multiple large randomized controlled trials (RCTs) and real-world studies have demonstrated the safety and efficacy of COVID-19 vaccines in the general population [6,7]. Most documented adverse effects associated with COVID-19 vaccines in individuals without underlying health conditions are generally of mild to moderate intensity and resolve within a short duration, typically lasting no more than a few days. Commonly reported side effects encompass localized pain at the site of injection, pyrexia, fatigue, cephalalgia, myalgia, rigors, and gastrointestinal disturbances. The likelihood of experiencing these side effects following vaccination is contingent upon the particular vaccine administered [8,9]. However, the safety of COVID-19 vaccination for some patients with specific diseases, especially autoimmune diseases, has not been confirmed. Because these patients often have immune dysfunction and are immunocompromised by long-term use of immunosuppressants, they are often excluded from vaccine RCTs participant selection [10].

Autoimmune neurological conditions (ANC) is a large group of autoimmune diseases characterized by an inappropriate immune response, in which the body mistakenly attacks the nervous system as an immune target [11], causing neurological damage, which often progresses or repeatedly exacerbates, resulting in disability or death. Common conditions include multiple sclerosis (MS), myasthenia gravis (MG), Guillain-Barre syndrome (GBS), neuromyelitis optica spectrum disorder (NMOSD), and chronic inflammatory demyelinating polyneuropathy (CIDP), etc. Infections are the most common cause of exacerbation in ANC [12], SARS-CoV-2 may also activate neuroinflammatory pathways [13], and severe pneumonia rates and mortality are higher in ANC patients infected with SARS-CoV-2 [14]. In addition, immunosuppressive therapy (IST) also increases the chance of severe pneumonia post-infection in ANC patients. For these reasons, vaccination is necessary to protect these patients from SARS-CoV-2 infection. Paradoxically, the vaccine contains weakened or inactivated parts (antigens) of specific organisms that can trigger immune responses and induce antibody production in the body [15]. In general, this attenuated version does not cause disease in healthy individuals, but has the potential to exacerbate autoimmune disease in patients with widespread immune dysregulation and immunodeficiency. The previous point of view is that inactivated vaccines are safe for ANC patients, and it has been supported by several studies on influenza vaccination 、human papillomavirus (HPV) vaccination and hepatitis B (HepB)vaccination in MG, MS and other diseases [[16], [17], [18]]. However, Since the onset of the COVID-19 pandemic, several studies have reported that vaccination induces autoimmune diseases or exacerbates pre-existing conditions [19,20]. Some other studies suggest that vaccination is safe for patients with neuroimmune diseases [5,21,22]. The differences may stem from the difference of sample size, type of disease, and type of vaccines. These contradictory conclusions fueled the hesitancy of patients with autoimmune diseases to vaccinate. In our previous survey on the vaccination of patients with myasthenia gravis, only 26.3 % of the patients were vaccinated, and most patients refused to vaccinate on the grounds of safety [23]. Therefore, in the context of the long-term epidemic of COVID-19, it is essential to clarify the safety of different types of ANC after vaccination with different types of vaccines. We selected several diseases with the highest incidence and most representative in ANC, including MS, MG, NMOSD, etc [24] and conducted a systematic review and a single-arm meta-analysis based on various eligible studies, to evaluate the safety of vaccination in patients with different diseases and different vaccine types. Aim to eliminate these patients' vaccination hesitancy through evidence-based medicine, and improve the protection rate of vulnerable populations.

## 2 Methods

This study is the first meta-data analysis to evaluate the safety of the COVID vaccine in ANC patients and conduct in the light of the Preferred Reporting Items for Systematic Reviews and Meta-Analyses (PRISMA) guidance [25].

### 2.1 Search strategy

The online literature search was conducted by two independent authors (FN and XC) from electronic databases, including PubMed, Embase, and Web of Science, from January 01, 2020, to December 31, 2022. The following sets of keywords were used for searching: Neuroimmune disorders, Central Nervous System Demyelinating Diseases, Multiple Sclerosis, Neuromyelitis Optica Spectrum Disorders, Myasthenia Gravis, Peripheral Nervous System Disease, chronic inflammatory demyelinating polyneuropathy combined using the operate ‘AND’ COVID 19 Vaccines, SARS Coronavirus 2 Vaccines or Vaccination (The detailed search strategy is provided in Supplementary Table 1.). The eligible titles were combined, and non–English-language titles and duplicates were removed. The titles and abstracts were reviewed independently by two reviewers (FN and XC)—full texts screen after the preliminary screening of relevant titles and abstracts. Ambiguity resolves after a discussion with a third reviewer (ZR).

### 2.2 Study selection

We included all associated observational studies about adverse events of COVID-19 vaccines in the systematic review. We included studies reporting at least one of the following outcomes in autoimmune neurological disorders, mentioned below.

### 2.3 Adverse events or side effects after SARS-CoV-2 vaccination in patients with autoimmune neurological disorders

#### 2.3.1 Exacerbation of underlying disorders after COVID-19 vaccination

We excluded the studies that have one or more of the following criteria: 1) Case, case series (the patient population of 5 participants or less), review, letter, conference abstract, guideline; 2) Studies on any species of vaccine other than SARS-CoV-2; 3) Studies that did not include ANC patients. We also screened full-text studies for vaccine efficacy to look for additional information regarding adverse events.

### 2.4 Data extraction

Data extraction and validity assessment by two researchers (FN and XC) independently screened and extracted data according to a predetermined proforma in Microsoft Excel Version 16, including the data that were removed irrespective of the type of vaccine or the number of vaccine doses. Data extraction and validity assessment by two researchers (FN and XC) independently screened and extracted data according to a predetermined proforma in Microsoft Excel Version 16, including 1) bibliometric information: title, first author, date of publication, and the country conducting the studies; 2)details of intervention: type of the vaccine, number of participants receiving each dose of vaccine; 3) demographic information: age, sex, and disease; 4) general methodological details: length of follow up; 5) outcome information: outcomes of safety and details of adverse events. The third researcher (ZR) resolved any disagreements during the process.

#### 2.4.1 Outcomes

A single-arm meta-analysis was performed because of the insufficiency of control arms—the pooled incidence rates of adverse events after the COVID-19 vaccine was calculated. The analysis was performed separately for several doses of the vaccine. We calculated the pooled incidence rates of adverse events for different types of ANC disease following vaccination. We also calculated the pooled rates of deterioration or recrudescence of underlying disease activity in ANC after COVID vaccination.

### 2.5 Data analysis

All analyses were conducted on R statistical software (version 4.2.2); in addition to the base package, the “meta” and “metafor” package was used [26]. We used the random effects model to calculate the pooled incidence rate with the inverse variance approach and the corresponding 95 % confidence intervals (CI) for the primary outcomes of interest. The I^2^ value represents the percentage of total variation among studies due to heterogeneity rather than chance. After that, the random effects models were used because of the underlying heterogeneity in the studies, containing types of neuroimmune disorders, vaccines, and doses, and the reported outcomes (I^2^ ≥ 50 % or p < 0.05). We performed subgroup analyses to ascertain if results were effect by the type and dose of vaccine or the different disease types. The forest plot was used to graphically represent the result of conducted subgroup meta-analysis. Publication bias was assessed using funnel plots and visual inspection for funnel plot asymmetry. Egger's test was used to assess publication bias. Sensitivity analysis was conducted to evaluate the robustness and reliability of the results.

## 3 Results

**Fig. 1:**
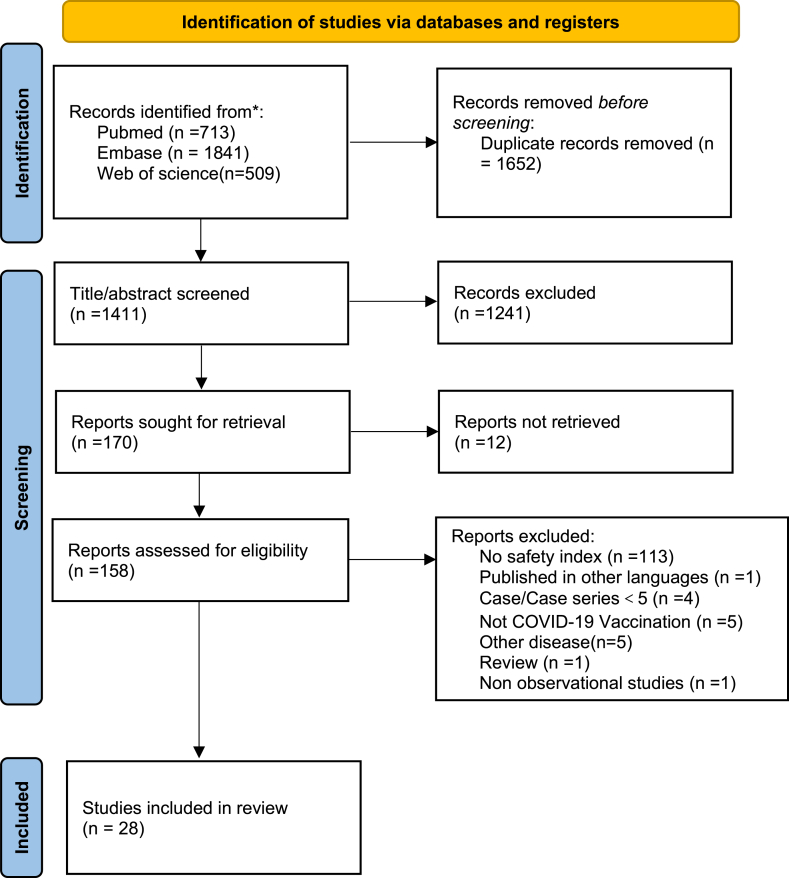
PRISMA flow chart depicting the study screening and selection of the systematic review.

As shown in Fig. 1 (PRISMA flow chart) summarizes the selection of studies. A total of 3063 citations were identified from databases, including PubMed, Web of Science, and Embase. There were 1652 duplicates. After screening the title and abstract, 1241 citations were removed. Full-text copies of twelve articles could not be achieved, and 158 full-text studies were finally reviewed. 113 studies were excluded due to no safety index, and detailed exclusion reasons for other studies are shown in figuer1. Eventually, 28 studies were included in the final meta-analysis. Studies characteristics of the selected are shown in Table 1 [[20], [21], [22],[27], [28], [29], [30], [31], [32], [33], [34], [35], [36], [37], [38], [39], [40], [41], [42], [43], [44], [45], [46], [47], [48], [49], [50], [51]].

**Table 1:** Characteristics of included studies.

Authors	country	Study design	Patients(n)	Mean age (SD) (years)	Type of ANC	Name of the vaccine	COVID-19 vaccine dosa(n)			Follow up days
							D1	D2	D3	
Anat Achiron	Israel	observational study	555		MS	mRNA vaccine	555	435		67
Mohammad Ali Sahraian	Iranian	observational study	579		MS	inactivated vaccines	579	350		21
Farren Basil Shaw Briggs		Retrospective, observational study	719	53.0 (11.8)	MS	mix	719	459		90
Jenna A	US	observational study	292	50.4 (12.4)	MS	mix				28
Fioravante Capone	Italy	Prospective，observational study	140	43.5 (12.7)	MS	mRNA vaccine	140	140		60
Ethel Ciampi	Chile	Multicentric, prospective, observational study	178	39.7 (11.2)	MS	mix	178			210
John R. Ciotti	US	prospective, observational, study	201		MS	mRNA vaccine	201			240
Massimiliano Di Filippo	Italy	Multicentric, prospective, observational study	324	42.7 (10.8)	MS	mRNA vaccine	324	322		60
Alessandro Dinoto	Italy	Multicentric, Retrospective, observational study	26		MS	mRNA vaccine	26	26		
Alon Doron	Israel	observational study	150	57.2 (18)	NMOSD	mRNA vaccine	150	147	133	42
Sapir Dreyer-Alster	Israel	prospective, observational, study	211		MG	mRNA vaccine			211	66
Masoud Etemadifar	Iran	Retrospective, observational study	517	37.81 (8.74)	MS	inactivated vaccines	517	417		
Antonio Farina	Italy	observational study	104		MS	mRNA vaccine	104	98	63	60
Josep Gamez MD	Spain	prospective, observational, study	91		MG	mRNA vaccine	91	89		225
Maria Pia Giannoccaro	Italy	longitudinal observational study	291		MS/MG	mRNA vaccine	300	300		
Vanja Jovicevic	Serbia	observational study	9	54.3 (10.3)	NMOSD	mix		9		
Trinchillo, A.	Italy	Retrospective, observational study	113	58.4 (15.5)	MG	mRNA vaccine			113	360
Stastna, D.	USA	Retrospective, observational study	1678		MS/NMOSD	mix	1661			90
Reyes-Leiva, D.	USA	Retrospective, observational study	100	55.85 (15.48)	MG	mRNA vaccine	100	100		90
Rauber, S.	Germany	Retrospective, observational study	59		MS	mix		59		28
Pincheira, A. U.	Australia	Retrospective observational study	200	64.3 (13.9)	MG	mix		200		14
Peric, S.	Serbia	cross-sectional study	125	61.7 (16.9)	MG	mix	87			365
Mariottini, A.	Italy	monocentric retrospective observational study	120		MS	mix				≥30
Maniscalco, G.T	Italy	Retrospective, observational study	310		MS	mRNA vaccine	310	288		180
Lotan, I.	Italy	Retrospective, observational study	55		MG	mRNA vaccine	55	51		168
Li, H Y	China	Retrospective, observational study	107	45.68 (1.49)	MG	inactivated vaccines	107	97	35	28
Kong, L.	China	Retrospective, observational study	187		MS/NMOSD					90
Kavosh, A	Iran	cross-sectional study	1538	40.45 (9.74)	MS	inactivated vaccines	1538	1538		

**Abbreviate:** ANC: autoimmune neurological conditions; MS: multiple sclerosis; MG: myasthenia gravis; NMOSD:neuromyelitis optica spectrum disorder; CI: confidence interval.

Of the 28 articles included in the final analysis, 31 cohort, 129,980 patients notified total adverse events after COVID-19 vaccination in patients with ANC. The diseases included were: MS (17/31, 54.8 %), MG (10/31, 32.3 %), and NMOSD (4/31, 12.9 %). Studies reported that the incidence of adverse reactions after the first, second, and third dose vaccination was 20(71.4 %), 17(60.7 %), and 6 (21.4 %), respectively. For different vaccine types, mRNA vaccines (either Pfizer-BioNTech or Moderna) accounted for 46.4 %, inactivated vaccines (CoronaVac, Sinovac) accounted for 14.3 %, and mixed vaccination types accounted for 39.3 %.

### 3.1 The incidence of post-vaccination adverse events in general

**Fig. 2:**
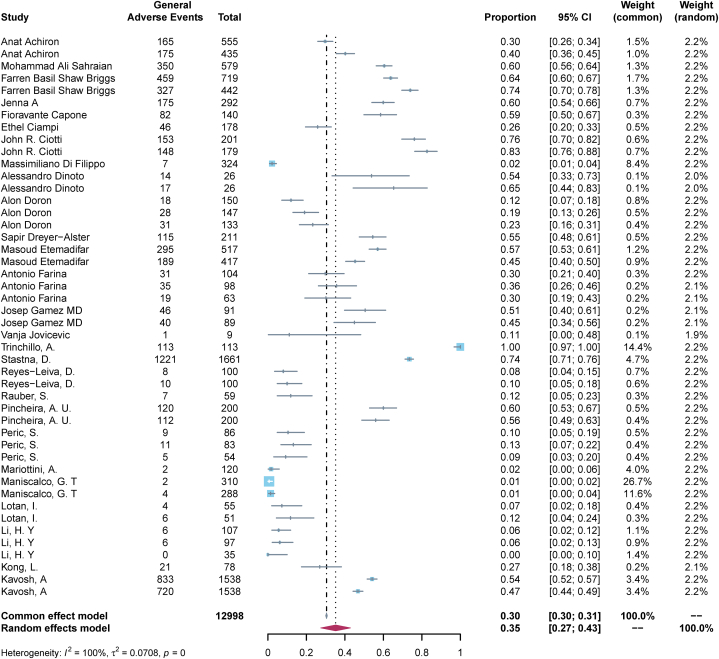
Forest maps of General adverse events following COVID-19 vaccination in patients with ANC.

The meta-analysis of the pooled incidence rate of general adverse events following post-vaccination, which contained 27 observational studies, is shown in Fig. 2. The pooled incidence rate of general adverse events in ANC patients following COVID-19 vaccination was 0.35 (95%CI, 0.27–0.43, I^2^ = 100 %). The most common type of adverse event was local injection reaction, with a pooled incidence rate of 0.27 (95 % CI, 0.18–0.36, I^2^ = 98 %); followed by fatigue 0.16 (95 % CI: 0.11–0.21, I^2^ = 93 %), weakness 0.15 (95 % CI: 0.00–0.31, I^2^ = 97 %), myalgia 0.13 (95 % CI: 0.08–0.19, I^2^ = 97 %), fever 0.11 (95 % CI: 0.07–0.15, I^2^ = 95 %), headache 0.11 (95 % CI: 0.07–0.16, I^2^ = 97 %), and chills 0.09 (95 % CI: 0.03–0.16, I^2^ = 96 %) **(**Supplementary Fig. 1**).**

### 3.2 The incidence of adverse events in subgroup

Subgroup analysis by disease type showed the highest total adverse event pooled incidence rate of patients with MS at 0.43 (95 % CI, 0.31–0.55, I^2^ = 100 %). MG and NMOSD of 0.26 (95 % CI, 0.15–0.37, I^2^ = 100 %) and 0.30 (95 % CI, 0.00–0.63, I^2^ = 91 %), respectively (Supplementary Fig. 2). The pooled incidence rate of adverse reactions following different immunization doses was the same, 0.37 (95 % CI, 0.25–0.49, I^2^ = 100 %) after the first dose, 0.34 (95 % CI, 0.21–0.47, I^2^ = 99 %) after the second dose and 0.36 (95 % CI, 0.27–0.44, I^2^ = 100 %) after the third dose (Supplementary Fig. 3). The pooled incidence rate of adverse events post-vaccination was similar across vaccine types, 0.35 (95 % CI, 0.23–0.47, I^2^ = 100 %) for mRNA vaccines, 0.34 (95 % CI, 0.13–0.56, I^2^ = 99 %) for inactivated vaccines, and 0.36 (95 % CI, 0.19–0.53, I^2^ = 100 %) for mixed vaccines (Supplementary Fig. 4).

### 3.3 The exacerbation pooled incidence rate of post-vaccination adverse events in different neuroimmune diseases

**Fig. 3:**
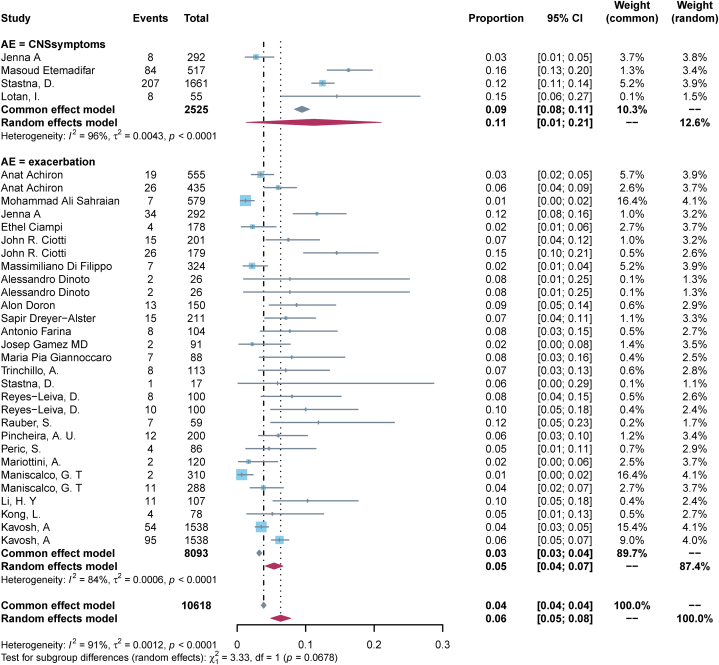
Top-panel: Forest maps of adverse events causing Neurological symptoms \in ANC patients following COVID-19 vaccination; **bottom panel:** Forest maps of causing exacerbation of disease following COVID-19 vaccination.

**Fig. 4:**
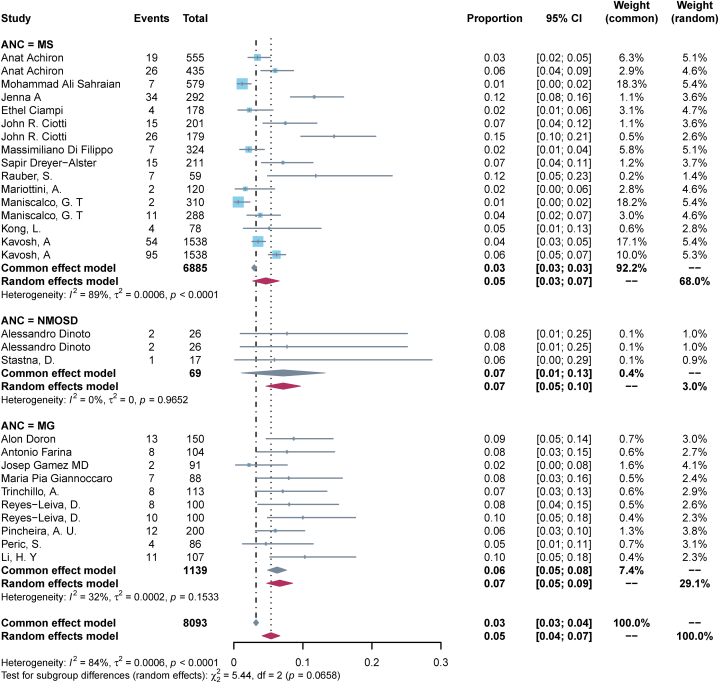
Forest maps of causing exacerbation of disease following COVID-19 vaccination with subtyped for type of ANC.

**Fig. 5:**
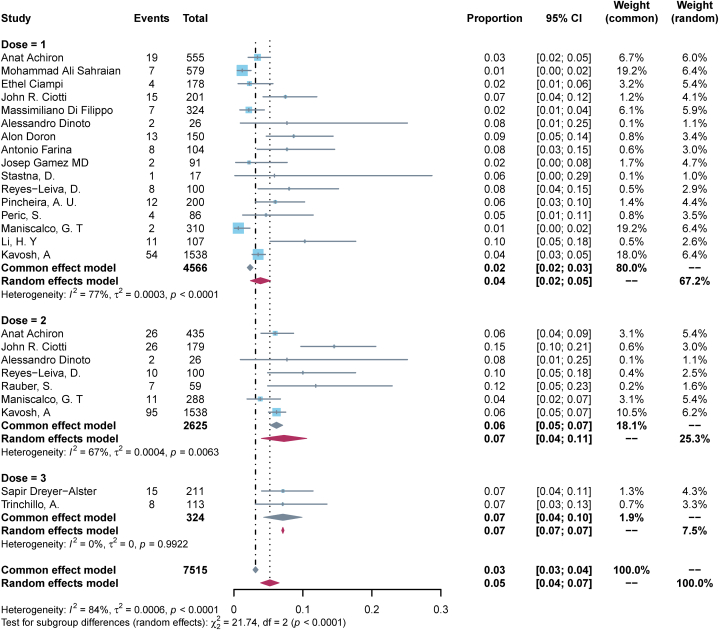
Forst maps of causing exacerbation of disease following COVID-19 vaccination with subtyped for dose of vaccine.

**Fig. 6:**
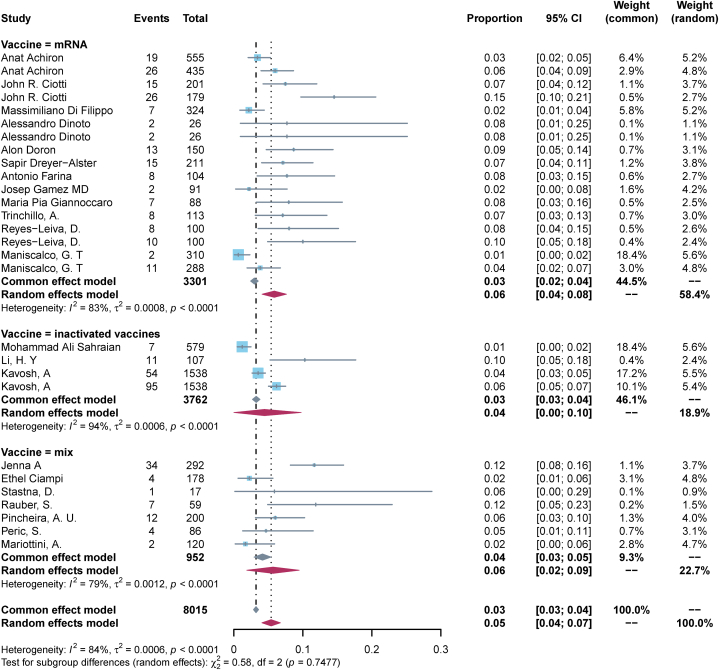
Forest maps of causing exacerbation of disease following COVID-19 vaccination with subtyped for type of vaccine.

23 (82.1 %) studies reported a worsening of pre-existing ANC post-vaccination. The pooled incidence rate of exacerbation of pre-existing disease in ANC patients following COVID-19 vaccination was calculated to be 0.05 (95%CI, 0.04–0.07, I^2^ = 84 %) (Fig. 3-bottom panel). Subgroup analysis by disease type showed that the pooled incidence rate of MS exacerbation after vaccination was the lowest at 0.05 (95 % CI, 0.03–0.07, I^2^ = 89 %), MG and NMOSD of 0.06 (95 % CI, 0.05–0.08, I^2^ = 32 %), and 0.07 (95 % CI, 0.01–0.13, I^2^ = 0 %), respectively (Fig. 4); Subgroup analysis by vaccination dose showed that the incidence of exacerbation of pre-existing disease was the lowest after the first dose, 0.04 (95 % CI, 0.02–0.05, I^2^ = 77 %), and was the same after the second dose and the third dose, 0.07 (95 % CI, 0.04–0.11, I^2^ = 67 %) and 0.07 (95 % CI, 0.04–0.10, I^2^ = 0 %) (Fig. 5); Subgroup analysis by vaccine type showed that the incidence of pre-existing disease exacerbations was 0.06 (95 % CI, 0.04–0.08, I^2^ = 83 %) after mRNA vaccination, 0.04 (95 % CI, 0.00–0.10, I^2^ = 94 %) for inactivated vaccines, and 0.06 (95 % CI, 0.02–0.09, I^2^ = 79 %) for mixed vaccines (Fig. 6). Four (14.3 %) studies reported post-vaccination neurologic adverse events, with a pooled incidence of 0.11 (95 % CI, 0.01–0.21, I^2^ = 96 %) (Fig. 3-Top Panel).

### 3.4 Risk of bias and sensitivity analysis

**Fig. 7:**
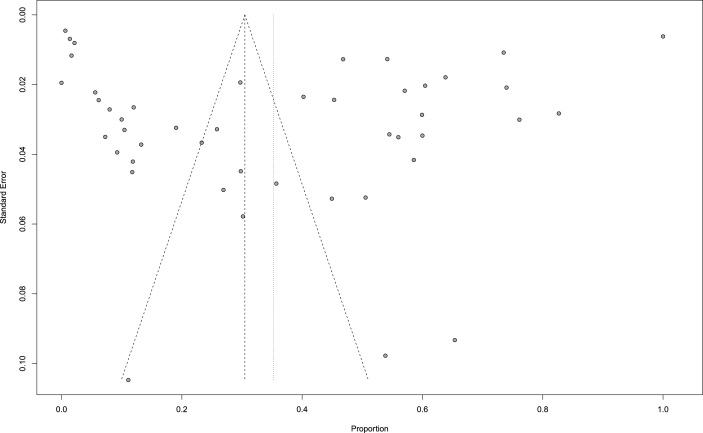
The funnel plot of included studies.

Visual inspection of the funnel plot provided no evidence of a significant publication bias (Fig. 7), confirmed by Egger's test for publication bias (P = 0.9008) (Supplementary Fig. 5). Leave one-out sensitivity analysis was carried out to assess the pooled incidence rate of adverse events. The results revealed that no study significantly impacted the overall effect (Supplementary Fig. 6).

## 4 Discussion

This study reviewed the safety studies of COVID-19 vaccination in patients with autoimmune neurological conditions and conducted a systematic review and meta-analysis. The results of the study showed that the pooled incidence rate of general adverse events after COVID-19 vaccination in ANC patients was similar to that in healthy people (35 %: 25%–38 %), vaccination had little impact on the underlying neurological autoimmune disease, the rate of disease exacerbation induced by the vaccine was meager (5 %), and COVID-19 vaccination was generally well tolerated by ANC patients.

Infection is an apparent predisposing or aggravating factor for autoimmune diseases, and it has been documented that COVID-19 infection induces neuroimmune diseases or leads to the aggravation of pre-existing conditions [52]. Patients with ANC are at increased risk of developing acute respiratory distress syndrome and multiple organ failure after being infected with the SARS-CoV-2 virus because of their immunosuppressed status [53]; therefore, protecting this group of patients is a priority, and some international consensus also recommends that people with neuroimmune diseases be vaccinated against SARS-CoV-2 as soon as possible [54].

However, there is a concern that vaccination may activate autoimmune responses. Theoretically, the vaccine may also trigger ANC or lead to deterioration or relapse of the disease in ANC patients. Although more than 5 billion people have been vaccinated worldwide, results from some observational studies show that there are still 10%–20 % of patients with neuroimmune diseases are hesitant to vaccinate [55]. There are many reasons for hesitation, Including religious beliefs, misconceptions about vaccines, distrust of vaccines, concerns about vaccine safety and effectiveness, and more. However, none of these arguments have any scientific basis and have been proven wrong [56]. Comprehensive vaccination and the establishment of population immunity barriers are the most promising measures to end the pandemic. At present, it is estimated that there are morez than 30 million patients with autoimmune diseases in the world [57]. Patients who have not been vaccinated may become a breakthrough in the immune barrier of the population and become the source of repeated epidemics of the virus. Therefore, a better understanding of the safety of COVID-19 vaccination in ANC patients is crucial to help improve vaccination, and it's also one of the keys to ending the pandemic.

After vaccination, we found the pooled incidence rate of general adverse events to be approximately 35 %. Most of the symptoms were characterized by transient post-vaccination adverse reactions, such as pain, swelling, redness, fever, chills, fatigue, headache, myalgia, and weakness at the injection site, and the incidence of these common adverse reactions was similar to that in healthy people [7]. Subgroup analysis of disease category showed that patients with multiple sclerosis had a slightly higher rate of general adverse events (43 %) than those with neuromyelitis optica and myasthenia gravis, but less than half overall. Due to the differences in the prevalence of different diseases among ethnic groups and regions, geographical differences in most conditions, and considerable heterogeneity between studies, the incidence of adverse events in the three common neuroimmune disorders cannot be directly compared. There was no significant difference in the incidence of general adverse events among all three conditions compared with that in healthy people similar incidence of general adverse events in all three diseases to that observed in healthy people, with no significant difference. Subgroup analysis of different vaccine vaccinations showed that the incidence of general adverse reactions caused by different doses was essentially the same for patients with neuroimmune diseases. Because the virus continuously mutates during transmission the virus constantly mutates during transmission, breakthrough infections occur at a high incidence in patients receiving regular doses of the vaccine, and many studies have shown that universal booster vaccination could be a current strategy to prevent breakthrough infections from COVID‐19 [58]. From the pooled analysis, there was no increase in the incidence of general adverse events in ANC patients who received three doses of vaccine there was no increase the incidence of general adverse reactions in patients with ANC who received three vaccinations, this finding suggests that providing booster doses to enhance the protective effect for ANC patients further could be a feasible strategy that could be considered that providing booster needles to enhance the protective effect of ANC patients further. Currently, the most widely used vaccine types worldwide are mRNA and inactivated. Subgroup analysis results show little overall difference in the incidence of general adverse events between these two vaccines, so it is reasonable to recommend these two types of vaccines to patients with neuroimmune diseases from a safety point of view.

Whether COVID‐19 vaccination exacerbates underlying neuroimmune diseases has been a controversial issue and a major cause of vaccination hesitancy in patients with neuroimmune diseases. A questionnaire survey on vaccination willingness conducted by KIM et al. among South Korean people with myasthenia gravis reported that patients who had experienced myasthenic crises were more resistant to vaccination in 160 questionnaire results, which may be related to their greater concern about the exacerbation of underlying diseases [59]. Yap, S. M. et al. Surveyed the willingness to vaccinate against COVID-19 among people with multiple sclerosis in Northern Ireland, and the results showed that about 20 % of MS patients are antipathetic to vaccines [60]. Most of this antipathy stems from concerns that vaccination may trigger or worsen the disease [61]. The association of vaccination with disease recurrence has been reported previously in several studies; a review incorporated 10 observational studies with a total of 1299 myasthenia gravis patients revealed that only 60 (4.26 %) patients developed an acute exacerbation of MG after vaccination [14]; In a study conducted in Kuwait, the probability of disease worsening and relapse following vaccination was 5.5 % and 1.8 %, respectively, in MS patients [5]. Giannoccaro et al. reported no difference in the relapse rate of NMOSD before and after vaccination [40]. In a multicenter prospective clinical study by Ad'aja E. Baars et al., 1152 patients with neurological autoimmune diseases, including GBS, CIDP, or multifocal motor neuropathy (MMN), were enrolled. The results showed that the exacerbation rates of these three underlying diseases after vaccination were 0 %, 3 %, and 4 %, respectively. Those studies suggest vaccination does not increase the risk of disease exacerbation and provides evidence for vaccination safety in patients with ANC [62]. Despite most studies indicating no significant worsening of pre-existing neurological and immunological conditions following vaccination, divergent outcomes exist due to variations in the disease under study, population inclusion criteria, sample size, geographic location, and vaccine type employed. Our meta-analysis demonstrates that the rate of recurrence or exacerbation of underlying diseases after SARS-CoV-2 vaccination is low, at approximately 7 %. Due to the lack of control groups in most vaccine studies, the proportion might be even lower, given that mass vaccination of the population may coincide with natural deterioration or relapse of the disease.

Furthermore, for patients with exacerbations of pre-existing conditions, most have a good prognosis, and there are almost no reports of severe deterioration (requiring adjustment of current treatment plans) reported, and this exacerbation is transient mainly after the treatment with steroids; patients can wholly or partially return to baseline values [27,43], Symptoms that exacerbated have also been reported to be self-limiting in some patients and recover without the need for any medical treatment [40]. In addition, we also found that the proportion of neurological adverse events caused by vaccination was relatively low (around 11 %), which further confirmed that vaccination had a minor impact on the course of ANC and that COVID-19 vaccination was safe for patients with neurological autoimmune diseases. Due to significant heterogeneity between studies, We conducted categorical subgroup analysis based on vaccination dose, vaccine type, and different ANC types to account for the considerable heterogeneity between studies.

### 4.1 Study limitations

There are some limitations to this study. Firstly, due to the unique nature of vaccine research, all the studies included had no control group. Although it was a single-arm meta-analysis, the meager pooled incidence rate can still reflect the vaccine's safety. Secondly, the overall heterogeneity in the analysis of the results of this study was extensive. Despite the use of a random-effects model, the presence of uncontrollable confounding factors may still exist. We tried to conduct multiple subgroup analyses stratified by confounding factors such as age, sex, comorbidities, etc., to test the stability of the results. Still, these factors could not be further adjusted due to insufficient raw data. We also performed bias analysis and sensitivity analysis to examine the influence of a specific study on the overall results, confirming that our included studies were extensive and of high quality and that individual studies had little effect on the overall results, so we judged the results to remain reliable. Thirdly, due to the significant variation in the number of publications for different diseases, we ultimately included only three types of conditions (MS, MG, NMOSD) after the screening, mainly because these diseases are rare and thus have fewer relevant studies available. Finally, most of the studies were conducted in 2022, during which the Omicron variant was dominant, and the incidence of adverse events may differ across different strains of the virus.

## 5 Conclusions

This study further confirms the safety of COVID-19 vaccination in the neuroimmune disease population, especially those with autoimmune neurological conditions. Patients with stable neuroimmune disorders should be encouraged to receive COVID-19 vaccination. Further research should monitor for serious adverse events following vaccination and the long-term impact of vaccination on patients with neuroimmune diseases.

## Data availability statement

The data from this study are available from the corresponding author upon reasonable request.

## Funding

This work was supported by the discipline innovation and development plan of Tangdu Hospital-major program of clinical research (Grant No. 2021LCYJ002), 10.13039/501100001809National Natural Science Foundation of China (Grant No. 82271378), 10.13039/501100012166National Key Research and Development Program (Grant No. 2022YFC3501304).

## CRediT authorship contribution statement

**Fan Ning:** Conceptualization, Data curation, Formal analysis, Methodology, Software, Validation, Visualization, Writing – original draft, Writing – review & editing. **Xiang-qi Cao:** Conceptualization, Data curation, Formal analysis, Software, Writing – original draft. **Qing-qing Wang:** Data curation. **Zhu-yi Li:** Data curation. **Zhe Ruan:** Data curation, Supervision, Writing – review & editing. **Ting Chang:** Conceptualization, Data curation, Formal analysis, Funding acquisition, Methodology, Software, Writing – review & editing.

## Declaration of competing interest

The authors declare that they have no known competing financial interests or personal relationships that could have appeared to influence the work reported in this paper.

## Abbreviations

ANC: autoimmune neurological conditions
SARS-CoV-2: severe acute respiratory syndrome type 2 coronavirus
RNA: ribonucleic acid
MS: multiple sclerosis
MG: myasthenia gravis
NMOSD: neuromyelitis optica spectrum disorder
GBS: Guillain-Barre syndrome
CIDP: chronic inflammatory demyelinating polyneuropathy
IST: immunosuppressive therapy
MMN: multifocal motor neuropathy
RCTs: randomized controlled trials
