# Supplementary figures and images for: Safety of SARS-CoV-2 vaccine in patients with autoimmune neurological conditions: A systematic review and meta-analysis
Source: Heliyon. 2023 Dec 23;10(1):e23944. doi: 10.1016/j.heliyon.2023.e23944 (PMC10796982; doi:10.1016/j.heliyon.2023.e23944)

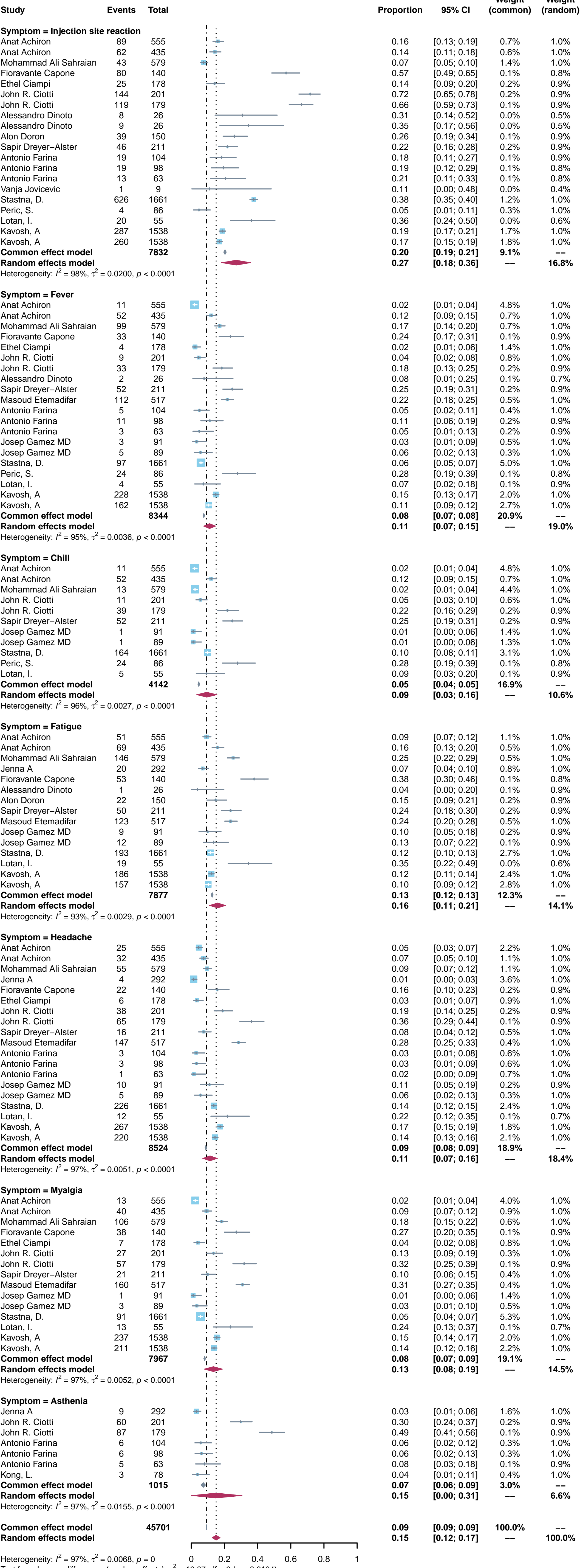

Supplement: Multimedia component 1 [file mmc1.pdf]

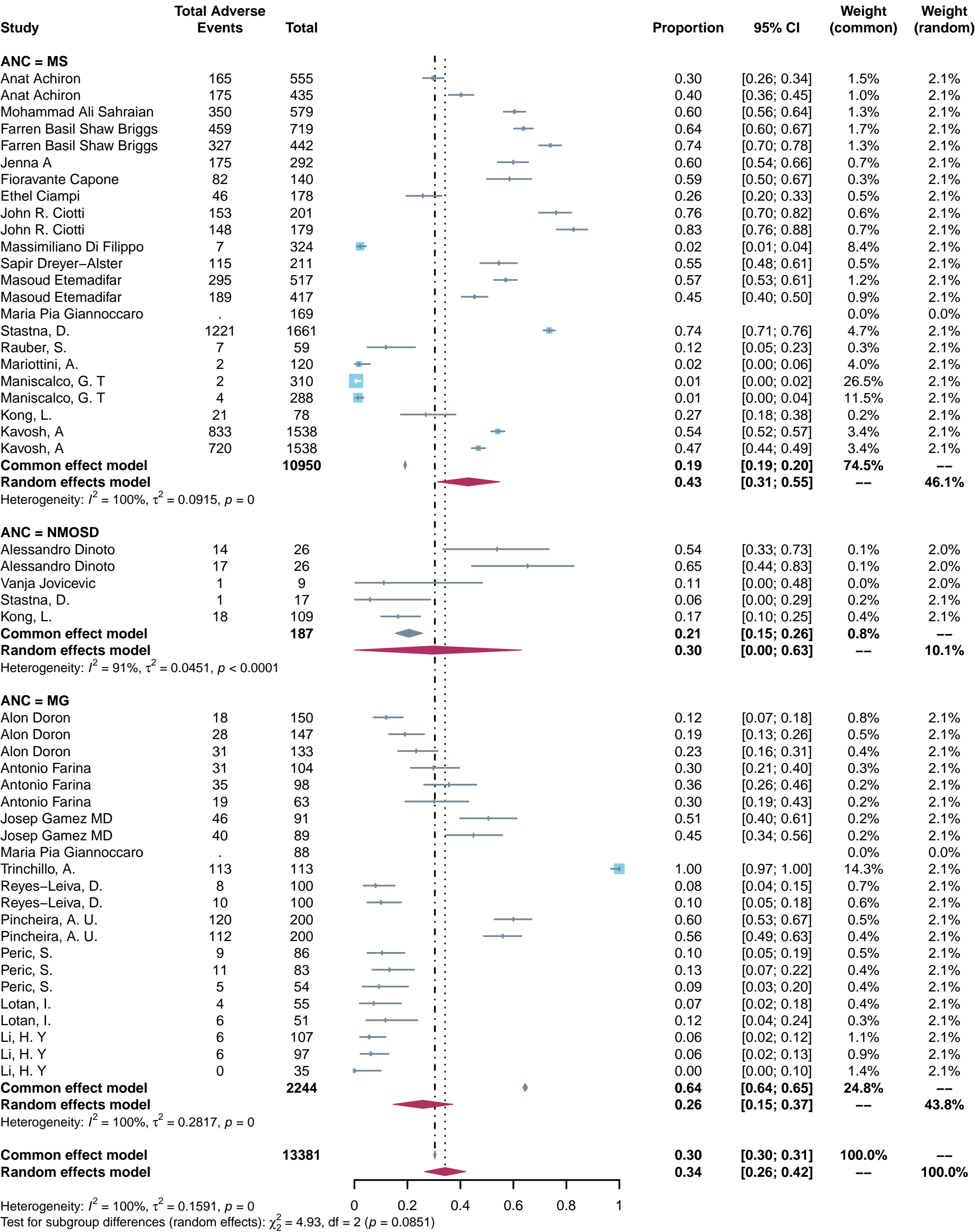

Supplement: Multimedia component 2 [file mmc2.pdf]

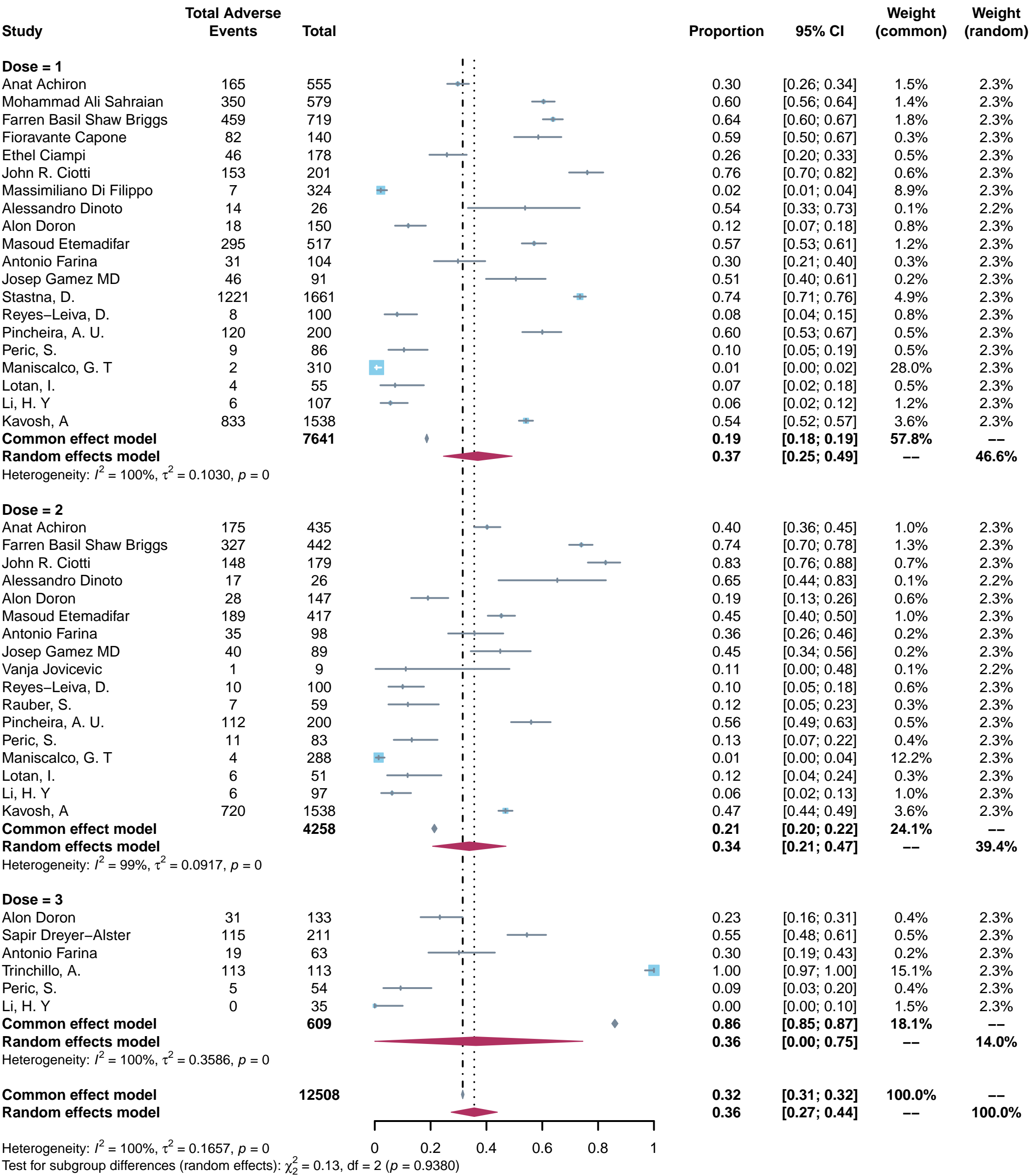

Supplement: Multimedia component 3 [file mmc3.pdf]

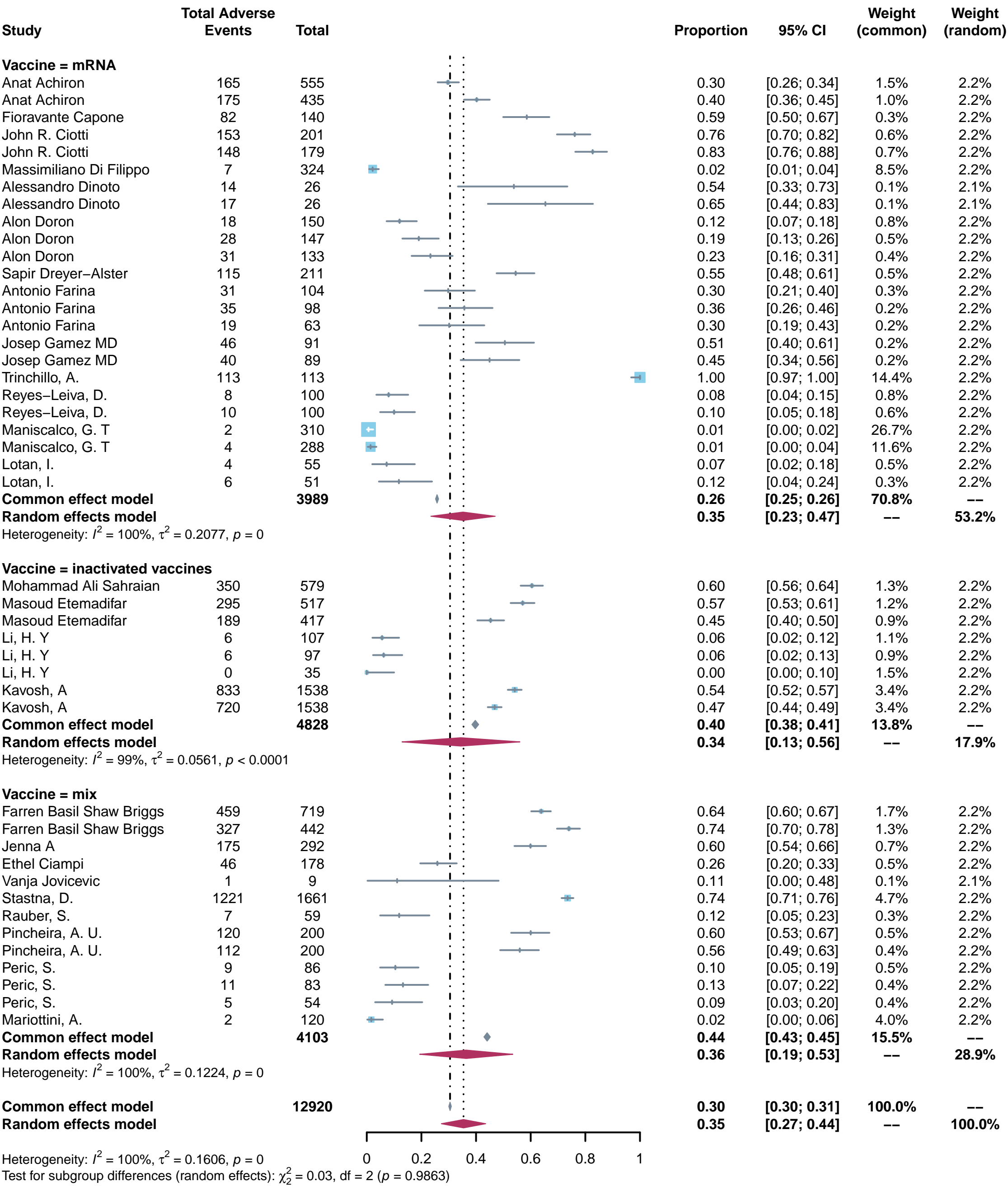

Supplement: Multimedia component 4 [file mmc4.pdf]

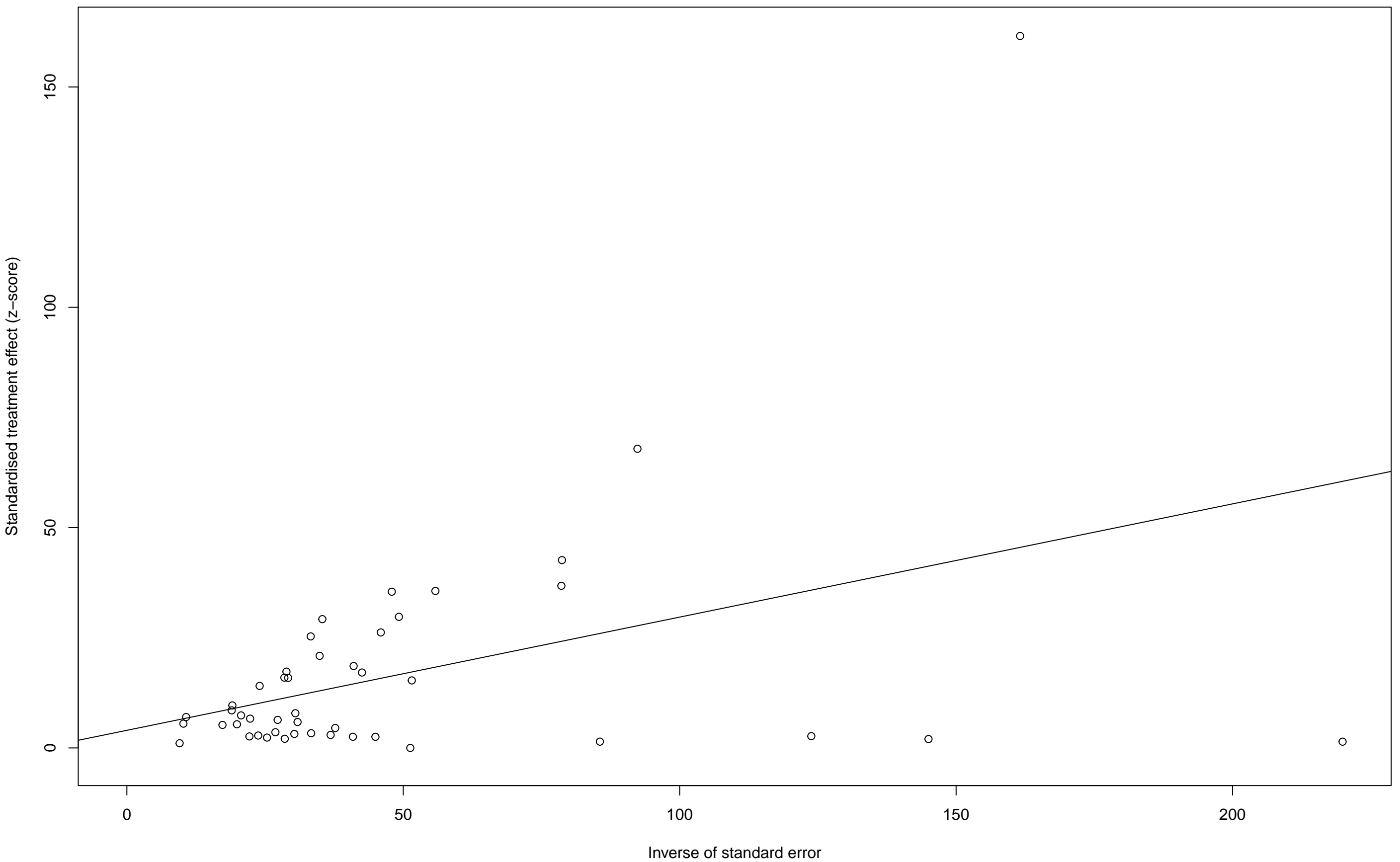

Supplement: Multimedia component 5 [file mmc5.pdf]

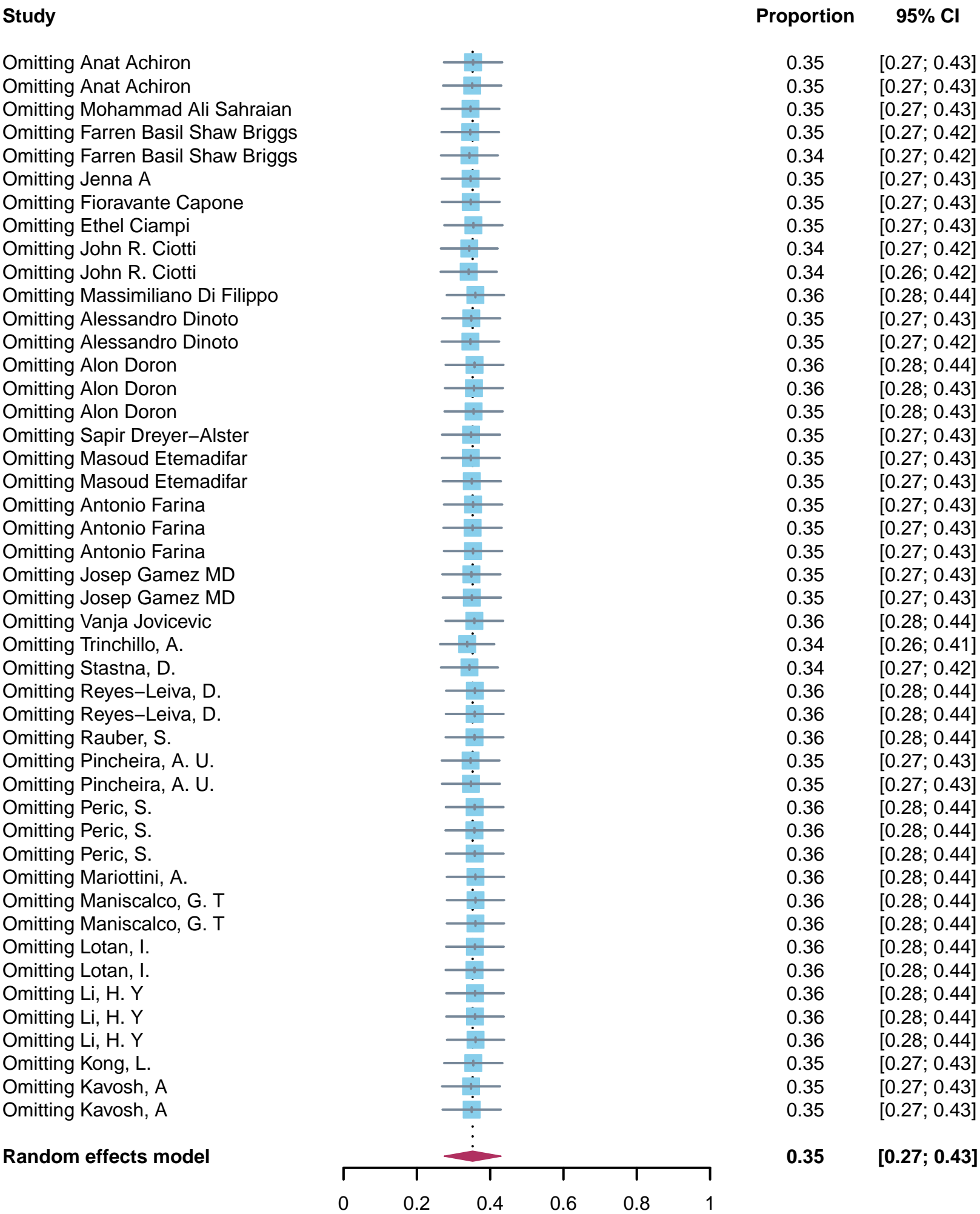

Supplement: Multimedia component 6 [file mmc6.pdf]
