# Supplementary material for: Safety of SARS-CoV-2 vaccine in patients with autoimmune neurological conditions: A systematic review and meta-analysis
Source: Heliyon. 2023 Dec 23;10(1):e23944. doi: 10.1016/j.heliyon.2023.e23944 (PMC10796982; doi:10.1016/j.heliyon.2023.e23944)
Supplement: Multimedia component 7 [file mmc7.docx]

Supplementary Table 1: Detailed search strategy for the systematic review

| PubMed | 31 December 2022 |  |
| --- | --- | --- |
| #1 | ((((((((((((((((((((((((((((((((((((((((((((((((((((COVID 19 Vaccines) OR (Vaccines, COVID-19)) OR (SARS Coronavirus 2 Vaccines)) OR (COVID19 Virus Vaccines)) OR (Vaccines, COVID19 Virus)) OR (Virus Vaccines, COVID19)) OR (COVID19 Virus Vaccine)) OR (Vaccine, COVID19 Virus)) OR (Virus Vaccine, COVID19)) OR (COVID19 Vaccines)) OR (Vaccines, COVID19)) OR (COVID19 Vaccine)) OR (Vaccine, COVID19)) OR (SARS-CoV-2 Vaccines)) OR (SARS CoV 2 Vaccines)) OR (Vaccines, SARS-CoV-2)) OR (SARS-CoV-2 Vaccine)) OR (SARS CoV 2 Vaccine)) OR (Vaccine, SARS-CoV-2)) OR (SARS2 Vaccines)) OR (Vaccines, SARS2)) OR (SARS2 Vaccine)) OR (Vaccine, SARS2)) OR (Coronavirus Disease 2019 Vaccines)) OR (Coronavirus Disease 2019 Vaccine)) OR (Coronavirus Disease 2019 Virus Vaccine)) OR (Coronavirus Disease 2019 Virus Vaccines)) OR (Coronavirus Disease-19 Vaccines)) OR (Coronavirus Disease 19 Vaccines)) OR (Vaccines, Coronavirus Disease-19)) OR (Coronavirus Disease-19 Vaccine)) OR (Coronavirus Disease 19 Vaccine)) OR (Vaccine, Coronavirus Disease-19)) OR (COVID 19 Vaccine)) OR (Vaccine, COVID 19)) OR (2019-nCoV Vaccine)) OR (2019 nCoV Vaccine)) OR (Vaccine, 2019-nCoV)) OR (2019 Novel Coronavirus Vaccines)) OR (2019 Novel Coronavirus Vaccine)) OR (2019-nCoV Vaccines)) OR (2019 nCoV Vaccines)) OR (Vaccines, 2019-nCoV)) OR (COVID-19 Vaccine)) OR (Vaccine, COVID-19)) OR (COVID-19 Virus Vaccines)) OR (COVID 19 Virus Vaccines)) OR (Vaccines, COVID-19 Virus)) OR (Virus Vaccines, COVID-19)) OR (COVID-19 Virus Vaccine)) OR (COVID 19 Virus Vaccine)) OR (Vaccine, COVID-19 Virus)) OR (Virus Vaccine, COVID-19) |  |
| #2 | (Neuroimmune disorders) |  |
| #1 AND #2 | (Neuroimmune disorders) AND (((((((((((((((((((((((((((((((((((((((((((((((((((((COVID 19 Vaccines) OR (Vaccines, COVID-19)) OR (SARS Coronavirus 2 Vaccines)) OR (COVID19 Virus Vaccines)) OR (Vaccines, COVID19 Virus)) OR (Virus Vaccines, COVID19)) OR (COVID19 Virus Vaccine)) OR (Vaccine, COVID19 Virus)) OR (Virus Vaccine, COVID19)) OR (COVID19 Vaccines)) OR (Vaccines, COVID19)) OR (COVID19 Vaccine)) OR (Vaccine, COVID19)) OR (SARS-CoV-2 Vaccines)) OR (SARS CoV 2 Vaccines)) OR (Vaccines, SARS-CoV-2)) OR (SARS-CoV-2 Vaccine)) OR (SARS CoV 2 Vaccine)) OR (Vaccine, SARS-CoV-2)) OR (SARS2 Vaccines)) OR (Vaccines, SARS2)) OR (SARS2 Vaccine)) OR (Vaccine, SARS2)) OR (Coronavirus Disease 2019 Vaccines)) OR (Coronavirus Disease 2019 Vaccine)) OR (Coronavirus Disease 2019 Virus Vaccine)) OR (Coronavirus Disease 2019 Virus Vaccines)) OR (Coronavirus Disease-19 Vaccines)) OR (Coronavirus Disease 19 Vaccines)) OR (Vaccines, Coronavirus Disease-19)) OR (Coronavirus Disease-19 Vaccine)) OR (Coronavirus Disease 19 Vaccine)) OR (Vaccine, Coronavirus Disease-19)) OR (COVID 19 Vaccine)) OR (Vaccine, COVID 19)) OR (2019-nCoV Vaccine)) OR (2019 nCoV Vaccine)) OR (Vaccine, 2019-nCoV)) OR (2019 Novel Coronavirus Vaccines)) OR (2019 Novel Coronavirus Vaccine)) OR (2019-nCoV Vaccines)) OR (2019 nCoV Vaccines)) OR (Vaccines, 2019-nCoV)) OR (COVID-19 Vaccine)) OR (Vaccine, COVID-19)) OR (COVID-19 Virus Vaccines)) OR (COVID 19 Virus Vaccines)) OR (Vaccines, COVID-19 Virus)) OR (Virus Vaccines, COVID-19)) OR (COVID-19 Virus Vaccine)) OR (COVID 19 Virus Vaccine)) OR (Vaccine, COVID-19 Virus)) OR (Virus Vaccine, COVID-19)) | 10 |
| #3 | ("central nervous system"[MeSH Terms] OR ("central"[All Fields] AND "nervous"[All Fields] AND "system"[All Fields]) OR "central nervous system"[All Fields]) AND ("demyelinating diseases"[MeSH Terms] OR ("demyelinating"[All Fields] AND "diseases"[All Fields]) OR "demyelinating diseases"[All Fields]) |  |
| #1 AND #3 | (((((((((((((((((((((((((((((((((((((((((((((((((((((COVID 19 Vaccines) OR (Vaccines, COVID-19)) OR (SARS Coronavirus 2 Vaccines)) OR (COVID19 Virus Vaccines)) OR (Vaccines, COVID19 Virus)) OR (Virus Vaccines, COVID19)) OR (COVID19 Virus Vaccine)) OR (Vaccine, COVID19 Virus)) OR (Virus Vaccine, COVID19)) OR (COVID19 Vaccines)) OR (Vaccines, COVID19)) OR (COVID19 Vaccine)) OR (Vaccine, COVID19)) OR (SARS-CoV-2 Vaccines)) OR (SARS CoV 2 Vaccines)) OR (Vaccines, SARS-CoV-2)) OR (SARS-CoV-2 Vaccine)) OR (SARS CoV 2 Vaccine)) OR (Vaccine, SARS-CoV-2)) OR (SARS2 Vaccines)) OR (Vaccines, SARS2)) OR (SARS2 Vaccine)) OR (Vaccine, SARS2)) OR (Coronavirus Disease 2019 Vaccines)) OR (Coronavirus Disease 2019 Vaccine)) OR (Coronavirus Disease 2019 Virus Vaccine)) OR (Coronavirus Disease 2019 Virus Vaccines)) OR (Coronavirus Disease-19 Vaccines)) OR (Coronavirus Disease 19 Vaccines)) OR (Vaccines, Coronavirus Disease-19)) OR (Coronavirus Disease-19 Vaccine)) OR (Coronavirus Disease 19 Vaccine)) OR (Vaccine, Coronavirus Disease-19)) OR (COVID 19 Vaccine)) OR (Vaccine, COVID 19)) OR (2019-nCoV Vaccine)) OR (2019 nCoV Vaccine)) OR (Vaccine, 2019-nCoV)) OR (2019 Novel Coronavirus Vaccines)) OR (2019 Novel Coronavirus Vaccine)) OR (2019-nCoV Vaccines)) OR (2019 nCoV Vaccines)) OR (Vaccines, 2019-nCoV)) OR (COVID-19 Vaccine)) OR (Vaccine, COVID-19)) OR (COVID-19 Virus Vaccines)) OR (COVID 19 Virus Vaccines)) OR (Vaccines, COVID-19 Virus)) OR (Virus Vaccines, COVID-19)) OR (COVID-19 Virus Vaccine)) OR (COVID 19 Virus Vaccine)) OR (Vaccine, COVID-19 Virus)) OR (Virus Vaccine, COVID-19)) AND (("central nervous system"[MeSH Terms] OR ("central"[All Fields] AND "nervous"[All Fields] AND "system"[All Fields]) OR "central nervous system"[All Fields]) AND ("demyelinating diseases"[MeSH Terms] OR ("demyelinating"[All Fields] AND "diseases"[All Fields]) OR "demyelinating diseases"[All Fields])) | 38 |
| #4 | ((((Sclerosis, Multiple) OR (Sclerosis, Disseminated)) OR (Disseminated Sclerosis)) OR (MS (Multiple Sclerosis))) OR (Multiple Sclerosis, Acute Fulminating) |  |
| #1 AND #4 | (((((((((((((((((((((((((((((((((((((((((((((((((((((COVID 19 Vaccines) OR (Vaccines, COVID-19)) OR (SARS Coronavirus 2 Vaccines)) OR (COVID19 Virus Vaccines)) OR (Vaccines, COVID19 Virus)) OR (Virus Vaccines, COVID19)) OR (COVID19 Virus Vaccine)) OR (Vaccine, COVID19 Virus)) OR (Virus Vaccine, COVID19)) OR (COVID19 Vaccines)) OR (Vaccines, COVID19)) OR (COVID19 Vaccine)) OR (Vaccine, COVID19)) OR (SARS-CoV-2 Vaccines)) OR (SARS CoV 2 Vaccines)) OR (Vaccines, SARS-CoV-2)) OR (SARS-CoV-2 Vaccine)) OR (SARS CoV 2 Vaccine)) OR (Vaccine, SARS-CoV-2)) OR (SARS2 Vaccines)) OR (Vaccines, SARS2)) OR (SARS2 Vaccine)) OR (Vaccine, SARS2)) OR (Coronavirus Disease 2019 Vaccines)) OR (Coronavirus Disease 2019 Vaccine)) OR (Coronavirus Disease 2019 Virus Vaccine)) OR (Coronavirus Disease 2019 Virus Vaccines)) OR (Coronavirus Disease-19 Vaccines)) OR (Coronavirus Disease 19 Vaccines)) OR (Vaccines, Coronavirus Disease-19)) OR (Coronavirus Disease-19 Vaccine)) OR (Coronavirus Disease 19 Vaccine)) OR (Vaccine, Coronavirus Disease-19)) OR (COVID 19 Vaccine)) OR (Vaccine, COVID 19)) OR (2019-nCoV Vaccine)) OR (2019 nCoV Vaccine)) OR (Vaccine, 2019-nCoV)) OR (2019 Novel Coronavirus Vaccines)) OR (2019 Novel Coronavirus Vaccine)) OR (2019-nCoV Vaccines)) OR (2019 nCoV Vaccines)) OR (Vaccines, 2019-nCoV)) OR (COVID-19 Vaccine)) OR (Vaccine, COVID-19)) OR (COVID-19 Virus Vaccines)) OR (COVID 19 Virus Vaccines)) OR (Vaccines, COVID-19 Virus)) OR (Virus Vaccines, COVID-19)) OR (COVID-19 Virus Vaccine)) OR (COVID 19 Virus Vaccine)) OR (Vaccine, COVID-19 Virus)) OR (Virus Vaccine, COVID-19)) AND (((((Sclerosis, Multiple) OR (Sclerosis, Disseminated)) OR (Disseminated Sclerosis)) OR (MS (Multiple Sclerosis))) OR (Multiple Sclerosis, Acute Fulminating)) | 337 |
| #5 | (((((((((((((((((((((((Neuromyelitis optica spectrum disorder) OR (NMO Spectrum Disorder)) OR (NMO Spectrum Disorders)) OR (Neuromyelitis Optica (NMO) Spectrum Disorder)) OR (Neuromyelitis Optica Spectrum Disorders)) OR (Devic Neuromyelitis Optica)) OR (Devic Neuromyelitis Opticas)) OR (Neuromyelitis Optica, Devic)) OR (Neuromyelitis Opticas, Devic)) OR (Devic's Disease)) OR (Devics Disease)) OR (Disease, Devic's)) OR (Devic Disease)) OR (Disease, Devic)) OR (Devic Syndrome)) OR (Syndrome, Devic)) OR (Devic's Syndrome)) OR (Devics Syndrome)) OR (Syndrome, Devic's)) OR (Devic's Neuromyelitis Optica)) OR (Devics Neuromyelitis Optica)) OR (Neuromyelitis Optica, Devic's)) OR (Neuromyelitis Optica Spectrum Disorder)) OR (Neuromyelitis Optica (NMO) Spectrum Disorders) |  |
| #1 AND #5 | (((((((((((((((((((((((((((((((((((((((((((((((((((((COVID 19 Vaccines) OR (Vaccines, COVID-19)) OR (SARS Coronavirus 2 Vaccines)) OR (COVID19 Virus Vaccines)) OR (Vaccines, COVID19 Virus)) OR (Virus Vaccines, COVID19)) OR (COVID19 Virus Vaccine)) OR (Vaccine, COVID19 Virus)) OR (Virus Vaccine, COVID19)) OR (COVID19 Vaccines)) OR (Vaccines, COVID19)) OR (COVID19 Vaccine)) OR (Vaccine, COVID19)) OR (SARS-CoV-2 Vaccines)) OR (SARS CoV 2 Vaccines)) OR (Vaccines, SARS-CoV-2)) OR (SARS-CoV-2 Vaccine)) OR (SARS CoV 2 Vaccine)) OR (Vaccine, SARS-CoV-2)) OR (SARS2 Vaccines)) OR (Vaccines, SARS2)) OR (SARS2 Vaccine)) OR (Vaccine, SARS2)) OR (Coronavirus Disease 2019 Vaccines)) OR (Coronavirus Disease 2019 Vaccine)) OR (Coronavirus Disease 2019 Virus Vaccine)) OR (Coronavirus Disease 2019 Virus Vaccines)) OR (Coronavirus Disease-19 Vaccines)) OR (Coronavirus Disease 19 Vaccines)) OR (Vaccines, Coronavirus Disease-19)) OR (Coronavirus Disease-19 Vaccine)) OR (Coronavirus Disease 19 Vaccine)) OR (Vaccine, Coronavirus Disease-19)) OR (COVID 19 Vaccine)) OR (Vaccine, COVID 19)) OR (2019-nCoV Vaccine)) OR (2019 nCoV Vaccine)) OR (Vaccine, 2019-nCoV)) OR (2019 Novel Coronavirus Vaccines)) OR (2019 Novel Coronavirus Vaccine)) OR (2019-nCoV Vaccines)) OR (2019 nCoV Vaccines)) OR (Vaccines, 2019-nCoV)) OR (COVID-19 Vaccine)) OR (Vaccine, COVID-19)) OR (COVID-19 Virus Vaccines)) OR (COVID 19 Virus Vaccines)) OR (Vaccines, COVID-19 Virus)) OR (Virus Vaccines, COVID-19)) OR (COVID-19 Virus Vaccine)) OR (COVID 19 Virus Vaccine)) OR (Vaccine, COVID-19 Virus)) OR (Virus Vaccine, COVID-19)) AND ((((((((((((((((((((((((Neuromyelitis optica spectrum disorder) OR (NMO Spectrum Disorder)) OR (NMO Spectrum Disorders)) OR (Neuromyelitis Optica (NMO) Spectrum Disorder)) OR (Neuromyelitis Optica Spectrum Disorders)) OR (Devic Neuromyelitis Optica)) OR (Devic Neuromyelitis Opticas)) OR (Neuromyelitis Optica, Devic)) OR (Neuromyelitis Opticas, Devic)) OR (Devic's Disease)) OR (Devics Disease)) OR (Disease, Devic's)) OR (Devic Disease)) OR (Disease, Devic)) OR (Devic Syndrome)) OR (Syndrome, Devic)) OR (Devic's Syndrome)) OR (Devics Syndrome)) OR (Syndrome, Devic's)) OR (Devic's Neuromyelitis Optica)) OR (Devics Neuromyelitis Optica)) OR (Neuromyelitis Optica, Devic's)) OR (Neuromyelitis Optica Spectrum Disorder)) OR (Neuromyelitis Optica (NMO) Spectrum Disorders)) | 37 |
| #6 | (((((((((((((Myasthenia Gravis, Ocular) OR (Ocular Myasthenia Gravis)) OR (Myasthenia Gravis, Generalized)) OR (Generalized Myasthenia Gravis)) OR (Muscle-Specific Receptor Tyrosine Kinase Myasthenia Gravis)) OR (Muscle Specific Receptor Tyrosine Kinase Myasthenia Gravis)) OR (Muscle-Specific Tyrosine Kinase Antibody Positive Myasthenia Gravis)) OR (Muscle Specific Tyrosine Kinase Antibody Positive Myasthenia Gravis)) OR (MuSK MG)) OR (MuSK Myasthenia Gravis)) OR (Myasthenia Gravis, MuSK)) OR (Anti-MuSK Myasthenia Gravis)) OR (Anti MuSK Myasthenia Gravis)) OR (Myasthenia Gravis, Anti-MuSK) |  |
| #1 AND #6 | (((((((((((((((((((((((((((((((((((((((((((((((((((((COVID 19 Vaccines) OR (Vaccines, COVID-19)) OR (SARS Coronavirus 2 Vaccines)) OR (COVID19 Virus Vaccines)) OR (Vaccines, COVID19 Virus)) OR (Virus Vaccines, COVID19)) OR (COVID19 Virus Vaccine)) OR (Vaccine, COVID19 Virus)) OR (Virus Vaccine, COVID19)) OR (COVID19 Vaccines)) OR (Vaccines, COVID19)) OR (COVID19 Vaccine)) OR (Vaccine, COVID19)) OR (SARS-CoV-2 Vaccines)) OR (SARS CoV 2 Vaccines)) OR (Vaccines, SARS-CoV-2)) OR (SARS-CoV-2 Vaccine)) OR (SARS CoV 2 Vaccine)) OR (Vaccine, SARS-CoV-2)) OR (SARS2 Vaccines)) OR (Vaccines, SARS2)) OR (SARS2 Vaccine)) OR (Vaccine, SARS2)) OR (Coronavirus Disease 2019 Vaccines)) OR (Coronavirus Disease 2019 Vaccine)) OR (Coronavirus Disease 2019 Virus Vaccine)) OR (Coronavirus Disease 2019 Virus Vaccines)) OR (Coronavirus Disease-19 Vaccines)) OR (Coronavirus Disease 19 Vaccines)) OR (Vaccines, Coronavirus Disease-19)) OR (Coronavirus Disease-19 Vaccine)) OR (Coronavirus Disease 19 Vaccine)) OR (Vaccine, Coronavirus Disease-19)) OR (COVID 19 Vaccine)) OR (Vaccine, COVID 19)) OR (2019-nCoV Vaccine)) OR (2019 nCoV Vaccine)) OR (Vaccine, 2019-nCoV)) OR (2019 Novel Coronavirus Vaccines)) OR (2019 Novel Coronavirus Vaccine)) OR (2019-nCoV Vaccines)) OR (2019 nCoV Vaccines)) OR (Vaccines, 2019-nCoV)) OR (COVID-19 Vaccine)) OR (Vaccine, COVID-19)) OR (COVID-19 Virus Vaccines)) OR (COVID 19 Virus Vaccines)) OR (Vaccines, COVID-19 Virus)) OR (Virus Vaccines, COVID-19)) OR (COVID-19 Virus Vaccine)) OR (COVID 19 Virus Vaccine)) OR (Vaccine, COVID-19 Virus)) OR (Virus Vaccine, COVID-19)) AND ((((((((((((((Myasthenia Gravis, Ocular) OR (Ocular Myasthenia Gravis)) OR (Myasthenia Gravis, Generalized)) OR (Generalized Myasthenia Gravis)) OR (Muscle-Specific Receptor Tyrosine Kinase Myasthenia Gravis)) OR (Muscle Specific Receptor Tyrosine Kinase Myasthenia Gravis)) OR (Muscle-Specific Tyrosine Kinase Antibody Positive Myasthenia Gravis)) OR (Muscle Specific Tyrosine Kinase Antibody Positive Myasthenia Gravis)) OR (MuSK MG)) OR (MuSK Myasthenia Gravis)) OR (Myasthenia Gravis, MuSK)) OR (Anti-MuSK Myasthenia Gravis)) OR (Anti MuSK Myasthenia Gravis)) OR (Myasthenia Gravis, Anti-MuSK)) | 64 |
| #7 | (((((((((((Peripheral Nervous System Disease) OR (PNS Diseases)) OR (PNS Disease)) OR (Peripheral Neuropathies)) OR (Neuropathy, Peripheral)) OR (Peripheral Neuropathy)) OR (PNS (Peripheral Nervous System) Diseases)) OR (Peripheral Nerve Diseases)) OR (Nerve Disease, Peripheral)) OR (Nerve Diseases, Peripheral)) OR (Peripheral Nerve Disease)) OR (Peripheral Nervous System Disorders) |  |
| #1 AND #7 | (((((((((((((((((((((((((((((((((((((((((((((((((((((COVID 19 Vaccines) OR (Vaccines, COVID-19)) OR (SARS Coronavirus 2 Vaccines)) OR (COVID19 Virus Vaccines)) OR (Vaccines, COVID19 Virus)) OR (Virus Vaccines, COVID19)) OR (COVID19 Virus Vaccine)) OR (Vaccine, COVID19 Virus)) OR (Virus Vaccine, COVID19)) OR (COVID19 Vaccines)) OR (Vaccines, COVID19)) OR (COVID19 Vaccine)) OR (Vaccine, COVID19)) OR (SARS-CoV-2 Vaccines)) OR (SARS CoV 2 Vaccines)) OR (Vaccines, SARS-CoV-2)) OR (SARS-CoV-2 Vaccine)) OR (SARS CoV 2 Vaccine)) OR (Vaccine, SARS-CoV-2)) OR (SARS2 Vaccines)) OR (Vaccines, SARS2)) OR (SARS2 Vaccine)) OR (Vaccine, SARS2)) OR (Coronavirus Disease 2019 Vaccines)) OR (Coronavirus Disease 2019 Vaccine)) OR (Coronavirus Disease 2019 Virus Vaccine)) OR (Coronavirus Disease 2019 Virus Vaccines)) OR (Coronavirus Disease-19 Vaccines)) OR (Coronavirus Disease 19 Vaccines)) OR (Vaccines, Coronavirus Disease-19)) OR (Coronavirus Disease-19 Vaccine)) OR (Coronavirus Disease 19 Vaccine)) OR (Vaccine, Coronavirus Disease-19)) OR (COVID 19 Vaccine)) OR (Vaccine, COVID 19)) OR (2019-nCoV Vaccine)) OR (2019 nCoV Vaccine)) OR (Vaccine, 2019-nCoV)) OR (2019 Novel Coronavirus Vaccines)) OR (2019 Novel Coronavirus Vaccine)) OR (2019-nCoV Vaccines)) OR (2019 nCoV Vaccines)) OR (Vaccines, 2019-nCoV)) OR (COVID-19 Vaccine)) OR (Vaccine, COVID-19)) OR (COVID-19 Virus Vaccines)) OR (COVID 19 Virus Vaccines)) OR (Vaccines, COVID-19 Virus)) OR (Virus Vaccines, COVID-19)) OR (COVID-19 Virus Vaccine)) OR (COVID 19 Virus Vaccine)) OR (Vaccine, COVID-19 Virus)) OR (Virus Vaccine, COVID-19)) AND ((((((((((((Peripheral Nervous System Disease) OR (PNS Diseases)) OR (PNS Disease)) OR (Peripheral Neuropathies)) OR (Neuropathy, Peripheral)) OR (Peripheral Neuropathy)) OR (PNS (Peripheral Nervous System) Diseases)) OR (Peripheral Nerve Diseases)) OR (Nerve Disease, Peripheral)) OR (Nerve Diseases, Peripheral)) OR (Peripheral Nerve Disease)) OR (Peripheral Nervous System Disorders)) | 208 |
| #8 | ((((((((((((((CIDP) OR (Polyneuropathy, Inflammatory Demyelinating, Chronic)) OR (Inflammatory Polyradiculopathy, Chronic)) OR (Chronic Inflammatory Polyradiculopathies)) OR (Chronic Inflammatory Polyradiculopathy)) OR (Inflammatory Polyradiculopathies, Chronic)) OR (Polyradiculopathies, Chronic Inflammatory)) OR (Polyradiculopathy, Chronic Inflammatory)) OR (Polyradiculoneuropathy, Chronic Inflammatory)) OR (Chronic Inflammatory Polyradiculoneuropathy)) OR (Chronic Inflammatory Polyradiculoneuropathies)) OR (Inflammatory Polyradiculoneuropathies, Chronic)) OR (Inflammatory Polyradiculoneuropathy, Chronic)) OR (Polyradiculoneuropathies, Chronic Inflammatory)) OR (Chronic Inflammatory Demyelinating Polyradiculoneuropathy) |  |
| #1 AND #8 | (((((((((((((((((((((((((((((((((((((((((((((((((((((COVID 19 Vaccines) OR (Vaccines, COVID-19)) OR (SARS Coronavirus 2 Vaccines)) OR (COVID19 Virus Vaccines)) OR (Vaccines, COVID19 Virus)) OR (Virus Vaccines, COVID19)) OR (COVID19 Virus Vaccine)) OR (Vaccine, COVID19 Virus)) OR (Virus Vaccine, COVID19)) OR (COVID19 Vaccines)) OR (Vaccines, COVID19)) OR (COVID19 Vaccine)) OR (Vaccine, COVID19)) OR (SARS-CoV-2 Vaccines)) OR (SARS CoV 2 Vaccines)) OR (Vaccines, SARS-CoV-2)) OR (SARS-CoV-2 Vaccine)) OR (SARS CoV 2 Vaccine)) OR (Vaccine, SARS-CoV-2)) OR (SARS2 Vaccines)) OR (Vaccines, SARS2)) OR (SARS2 Vaccine)) OR (Vaccine, SARS2)) OR (Coronavirus Disease 2019 Vaccines)) OR (Coronavirus Disease 2019 Vaccine)) OR (Coronavirus Disease 2019 Virus Vaccine)) OR (Coronavirus Disease 2019 Virus Vaccines)) OR (Coronavirus Disease-19 Vaccines)) OR (Coronavirus Disease 19 Vaccines)) OR (Vaccines, Coronavirus Disease-19)) OR (Coronavirus Disease-19 Vaccine)) OR (Coronavirus Disease 19 Vaccine)) OR (Vaccine, Coronavirus Disease-19)) OR (COVID 19 Vaccine)) OR (Vaccine, COVID 19)) OR (2019-nCoV Vaccine)) OR (2019 nCoV Vaccine)) OR (Vaccine, 2019-nCoV)) OR (2019 Novel Coronavirus Vaccines)) OR (2019 Novel Coronavirus Vaccine)) OR (2019-nCoV Vaccines)) OR (2019 nCoV Vaccines)) OR (Vaccines, 2019-nCoV)) OR (COVID-19 Vaccine)) OR (Vaccine, COVID-19)) OR (COVID-19 Virus Vaccines)) OR (COVID 19 Virus Vaccines)) OR (Vaccines, COVID-19 Virus)) OR (Virus Vaccines, COVID-19)) OR (COVID-19 Virus Vaccine)) OR (COVID 19 Virus Vaccine)) OR (Vaccine, COVID-19 Virus)) OR (Virus Vaccine, COVID-19)) AND (((((((((((((((CIDP) OR (Polyneuropathy, Inflammatory Demyelinating, Chronic)) OR (Inflammatory Polyradiculopathy, Chronic)) OR (Chronic Inflammatory Polyradiculopathies)) OR (Chronic Inflammatory Polyradiculopathy)) OR (Inflammatory Polyradiculopathies, Chronic)) OR (Polyradiculopathies, Chronic Inflammatory)) OR (Polyradiculopathy, Chronic Inflammatory)) OR (Polyradiculoneuropathy, Chronic Inflammatory)) OR (Chronic Inflammatory Polyradiculoneuropathy)) OR (Chronic Inflammatory Polyradiculoneuropathies)) OR (Inflammatory Polyradiculoneuropathies, Chronic)) OR (Inflammatory Polyradiculoneuropathy, Chronic)) OR (Polyradiculoneuropathies, Chronic Inflammatory)) OR (Chronic Inflammatory Demyelinating Polyradiculoneuropathy)) | 19 |
| Embase | 31 December 2022 |  |
| #1 | 'SARS-COV-2 vaccine'/exp | 29581 |
| #2 | 'covid 19 vaccines '/exp OR ' covid 19 vaccines ' OR(( 'covid'/exp OR covid)AND19AND('vaccines'/exp OR vaccines) | 42680 |
| #3 | #1OR#2 | 42680 |
| #4 | 'neuroimmune disorders' OR(neuroimmune AND ('disorders'/exp disorder)) | 8919 |
| #3AND#4 |  | 22 |
| #5 | 'demyelinating disease'/exp | 208666 |
| #6 | 'central nervous system demyelinating diseases' OR (centrallexp OR central) AND nervous AND system AND demyelinating AND(diseaseslexp ORdiseases)) | 12713 |
| #7 | #5AND#6 | 20626 |
| #3AND#7 |  | 820 |
| #8 | 'sclerosis, multiple'/exp OR 'sclerosis, multiple'OR('sclerosis,'lexp OR sclerosis,)AND multiple) | 189526 |
| #3AND#8 |  | 699 |
| #9 | 'neuromyelitis optica spectrum disorderlexp OR 'neuromyelitis optica spectrum disorder' OR (/neuromyelitis AND optica AND ('spectrum'lexp ORspectrum)AND ('disorder'/exp OR disorder)) | 12167 |
| #3AND#9 |  | 88 |
| #10 | 'myasthenia gravis'lexp OR 'myasthenia gravis' OR('myasthenia'exp OR myasthenia)AND gravis) | 29898 |
| #3AND#10 |  | 84 |
| #11 | 'peripheral nervous system disease' OR (peripheral AND nervous AND system AND('disease"lexp OR disease)) | 126,100 |
| #3AND#11 |  | 79 |
| #12 | 'chronic inflammatory demyelinating polyneuropathytlexp OR 'chronic inflammatory demyelinating polyneuropathy' OR (chronic AND infammatory AND  demyelinating AND ('polyneuropathy'/exp OR polyneuropathy)) | 6,835 |
| #3AND#12 |  | 49 |
| Web of science | 31 December 2022 |  |
| #1 | (((((((((ALL=(COVID 19 Vaccines)) OR ALL=(Vaccines, COVID-19)) OR ALL=(SARS Coronavirus 2 Vaccines)) OR ALL=(COVID19 Virus Vaccines)) OR ALL=(Vaccines, COVID19 Virus)) OR ALL=(Virus Vaccines, COVID19)) OR ALL=(COVID19 Virus Vaccine)) OR ALL=(Vaccine, COVID19 Virus)) OR ALL=(Virus Vaccine, COVID19)) OR ALL=(COVID19 Vaccines) | 33915 |
| #2 | ALL=(Neuroimmune disorders) | 2161 |
| #1AND#2 |  | 3 |
| #3 | ALL=(Central Nervous System Demyelinating Diseases) | 6573 |
| #1AND#3 |  | 18 |
| #4 | ((((ALL=(Sclerosis, Multiple)) OR ALL=(Sclerosis, Disseminated)) OR ALL=(Disseminated Sclerosis)) OR ALL=(MS (Multiple Sclerosis))) OR ALL=(Multiple Sclerosis, Acute Fulminating) | 167952 |
| #1AND#4 |  | 355 |
| #5 | ((((((((((((((((((((((ALL=(Neuromyelitis optica spectrum disorder)) OR ALL=(NMO Spectrum Disorder)) OR ALL=(NMO Spectrum Disorders)) OR ALL=(Neuromyelitis Optica (NMO) Spectrum Disorder)) OR ALL=(Neuromyelitis Optica Spectrum Disorders)) OR ALL=(Devic Neuromyelitis Optica)) OR ALL=(Neuromyelitis Optica, Devic)) OR ALL=(Neuromyelitis Opticas, Devic)) OR ALL=(Devic's Disease)) OR ALL=(Devics Disease)) OR ALL=(Disease, Devic's)) OR ALL=(Devic Disease)) OR ALL=(Disease, Devic)) OR ALL=(Devic Syndrome)) OR ALL=(Syndrome, Devic)) OR ALL=(Devic's Syndrome)) OR ALL=(Devics Syndrome)) OR ALL=(Syndrome, Devic's)) OR ALL=(Devic's Neuromyelitis Optica)) OR ALL=(Devics Neuromyelitis Optica)) OR ALL=(Neuromyelitis Optica, Devic's)) OR ALL=(Neuromyelitis Optica Spectrum Disorder)) OR ALL=(Neuromyelitis Optica (NMO) Spectrum Disorders) | 5389 |
| #1AND#5 |  | 27 |
| #6 | (((((((((((((ALL=(Myasthenia Gravis, Ocular)) OR ALL=(Ocular Myasthenia Gravis)) OR ALL=(Myasthenia Gravis, Generalized)) OR ALL=(Generalized Myasthenia Gravis)) OR ALL=(Muscle-Specific Receptor Tyrosine Kinase Myasthenia Gravis)) OR ALL=(Muscle Specific Receptor Tyrosine Kinase Myasthenia Gravis)) OR ALL=(Muscle-Specific Tyrosine Kinase Antibody Positive Myasthenia Gravis)) OR ALL=(Muscle Specific Tyrosine Kinase Antibody Positive Myasthenia Gravis)) OR ALL=(MuSK MG)) OR ALL=(MuSK Myasthenia Gravis)) OR ALL=(Myasthenia Gravis, MuSK)) OR ALL=(Anti-MuSK Myasthenia Gravis)) OR ALL=(Anti MuSK Myasthenia Gravis)) OR ALL=(Myasthenia Gravis, Anti-MuSK) | 3338 |
| #1AND#6 |  | 17 |
| #7 | (((((((((((ALL=(Peripheral Nervous System Disease)) OR ALL=(PNS Diseases)) OR ALL=(PNS Disease)) OR ALL=(Peripheral Neuropathies)) OR ALL=(Neuropathy, Peripheral)) OR ALL=(Peripheral Neuropathy)) OR ALL=(PNS (Peripheral Nervous System) Diseases)) OR ALL=(Peripheral Nerve Diseases)) OR ALL=(Nerve Disease, Peripheral)) OR ALL=(Nerve Diseases, Peripheral)) OR ALL=(Peripheral Nerve Disease)) OR ALL=(Peripheral Nervous System Disorders) | 77452 |
| #1AND#7 |  | 71 |
| #8 | ((((((((((((((ALL=(CIDP)) OR ALL=(Polyneuropathy, Inflammatory Demyelinating, Chronic)) OR ALL=(Inflammatory Polyradiculopathy, Chronic)) OR ALL=(Chronic Inflammatory Polyradiculopathies)) OR ALL=(Chronic Inflammatory Polyradiculopathy)) OR ALL=(Inflammatory Polyradiculopathies, Chronic)) OR ALL=(Polyradiculopathies, Chronic Inflammatory)) OR ALL=(Polyradiculopathy, Chronic Inflammatory)) OR ALL=(Polyradiculoneuropathy, Chronic Inflammatory)) OR ALL=(Chronic Inflammatory Polyradiculoneuropathy)) OR ALL=(Chronic Inflammatory Polyradiculoneuropathies)) OR ALL=(Inflammatory Polyradiculoneuropathies, Chronic)) OR ALL=(Inflammatory Polyradiculoneuropathy, Chronic)) OR ALL=(Polyradiculoneuropathies, Chronic Inflammatory)) OR ALL=(Chronic Inflammatory Demyelinating Polyradiculoneuropathy) | 5425 |
| #1AND#7 |  | 18 |
